# NAP1L5 Promotes Nucleolar Hypertrophy and Is Required for Translation Activation During Cardiomyocyte Hypertrophy

**DOI:** 10.3389/fcvm.2021.791501

**Published:** 2021-12-17

**Authors:** Ningning Guo, Di Zheng, Jiaxin Sun, Jian Lv, Shun Wang, Yu Fang, Zhenyi Zhao, Sai Zeng, Qiuxiao Guo, Jingjing Tong, Zhihua Wang

**Affiliations:** ^1^Department of Cardiology, Renmin Hospital of Wuhan University, Wuhan, China; ^2^Shenzhen Key Laboratory of Cardiovascular Disease, Fuwai Hospital, Chinese Academy of Medical Sciences, Shenzhen, China; ^3^State Key Laboratory of Cardiovascular Disease, Fuwai Hospital, National Center for Cardiovascular Disease, Chinese Academy of Medical Sciences and Peking Union Medical College, Beijing, China; ^4^Health Science Center, School of Pharmacy, Shenzhen University, Shenzhen, China; ^5^School of Life Sciences, Central China Normal University, Wuhan, China

**Keywords:** cardiomyocyte hypertrophy, NAP1L5, translation control, nucleolar hypertrophy, ribosome assembly

## Abstract

Pathological growth of cardiomyocytes during hypertrophy is characterized by excess protein synthesis; however, the regulatory mechanism remains largely unknown. Using a neonatal rat ventricular myocytes (NRVMs) model, here we find that the expression of nucleosome assembly protein 1 like 5 (Nap1l5) is upregulated in phenylephrine (PE)-induced hypertrophy. Knockdown of Nap1l5 expression by siRNA significantly blocks cell size enlargement and pathological gene induction after PE treatment. In contrast, Adenovirus-mediated Nap1l5 overexpression significantly aggravates the pro-hypertrophic effects of PE on NRVMs. RNA-seq analysis reveals that Nap1l5 knockdown reverses the pro-hypertrophic transcriptome reprogramming after PE treatment. Whereas, immune response is dominantly enriched in the upregulated genes, oxidative phosphorylation, cardiac muscle contraction and ribosome-related pathways are remarkably enriched in the down-regulated genes. Although Nap1l5-mediated gene regulation is correlated with PRC2 and PRC1, Nap1l5 does not directly alter the levels of global histone methylations at K4, K9, K27 or K36. However, puromycin incorporation assay shows that Nap1l5 is both necessary and sufficient to promote protein synthesis in cardiomyocyte hypertrophy. This is attributable to a direct regulation of nucleolus hypertrophy and subsequent ribosome assembly. Our findings demonstrate a previously unrecognized role of Nap1l5 in translation control during cardiac hypertrophy.

## Introduction

The heart develops ventricular hypertrophy to compensate for increased hemodynamic workload under various stresses, and progressively leading to heart failure (1, 2). Cardiac hypertrophy is characterized by enlarged cell size and excess protein synthesis (3–5). Protein synthesis efficiency is fine-tuned at multiple steps, such as ribosomal gene transcription, ribosome assembly, ribosome export, and translation initiation, elongation and termination (6, 7). How translation activity is regulated during cardiac hypertrophy remains largely unexplored.

Primary regulation of translation occurs in nucleolus, where ribosomal DNA is transcribed and ribosome subunits are assembled. Cardiac hypertrophy is one of the first diseases identified to be associated with ribosomal DNA transcriptional disorders (8, 9). Accelerated polymerase I (PolI) transcription rate increases ribosome numbers during the development of cardiac hypertrophy (10). Upstream binding transcription factor (UBTF) has been shown to regulate PolI transcription activity in cardiac hypertrophy (11, 12). Despite these earlier observations, however, little progression has been made about translation control in heart diseases.

The mechanistic target of rapamycin (mTOR) plays a central role in protein synthesis by phosphorylating 70 kD ribosomal protein S6 kinase 1 (S6K1) and eukaryotic translation initiation factor 4E (eIF4E)-binding protein-1 (4E-BP1), which subsequently initiate a series of signal transduction to promote the operation of ribosomes (13, 14). Though generally thought to positively correlate with the pathogenesis of cardiac hypertrophy, there are numerous mysteries about the regulation and function of mTOR. Cardiac-specific ablation of raptor, the core component of mTOR complex 1 (mTORC1), impairs adaptive hypertrophy, but causes heart failure in mice (15). Simultaneous knockout of two genes encoding for S6K1, Rps6kb1, and Rps6kb2, has no effect on pressure overload-induced cardiac hypertrophy in mice (16). Interestingly, our previous study implicated that the activation of mTOR signaling is usually transient, and quickly fades out after hypertrophic stimulation (17). To what extent do the mTOR-dependent and the mTOR-independent mechanisms contribute to translational regulation during cardiac hypertrophy remains under question.

Nucleosome assembly protein 1 like (NAP1L) protein family has been identified as evolutionarily conserved histone chaperones assisting the assembly of nucleosomes with different histone variants (18–20). It consists of five members, namely NAP1L1-5, among which NAP1L1 is the member being firstly identified and best functionally characterized (21–23). In addition to a role in assisting H2A-H2B dimer incorporation into nucleosome, NAP1L1 also participates in gene expression regulation through epigenetic mechanisms (24, 25). Recently, NAP1L family members have been found to be functionally involved in cell proliferation and differentiation during development and human diseases, such as carcinoma and virus infection (26–40). In contrast, our knowledge about other family members is still limited. NAP1L5 is the newest member firstly identified in human liver malignancy as an imprinted gene (41, 42). Chang et al. found that the gene coding for NAP1L5 was hypomethylated in congenital heart diseases (43). However, the molecular function and the role of NAP1L5 in human diseases remains largely unknown.

Here we find that NAP1L5 expression is significantly upregulated in phenylephrine (PE)-induced cardiomyocyte hypertrophy. siRNA-mediated knockdown and adenovirus-mediated overexpression experiments suggest that NAP1L5 is required for the development of cardiomyocyte hypertrophy. Transcriptome analysis and puromycin incorporation assay reveal a crucial regulation of nucleolus hypertrophy, ribosome assembly and protein synthesis rate by NAP1L5. Our findings demonstrate a novel role of NAP1L5 in translation control during the pathological growth of cardiomyocytes, and provide potential molecular targets to treat cardiac hypertrophy.

## Results

### NAP1L5 Silence Abolishes PE-Induced Cardiomyocyte Hypertrophy

**Figure 1:**
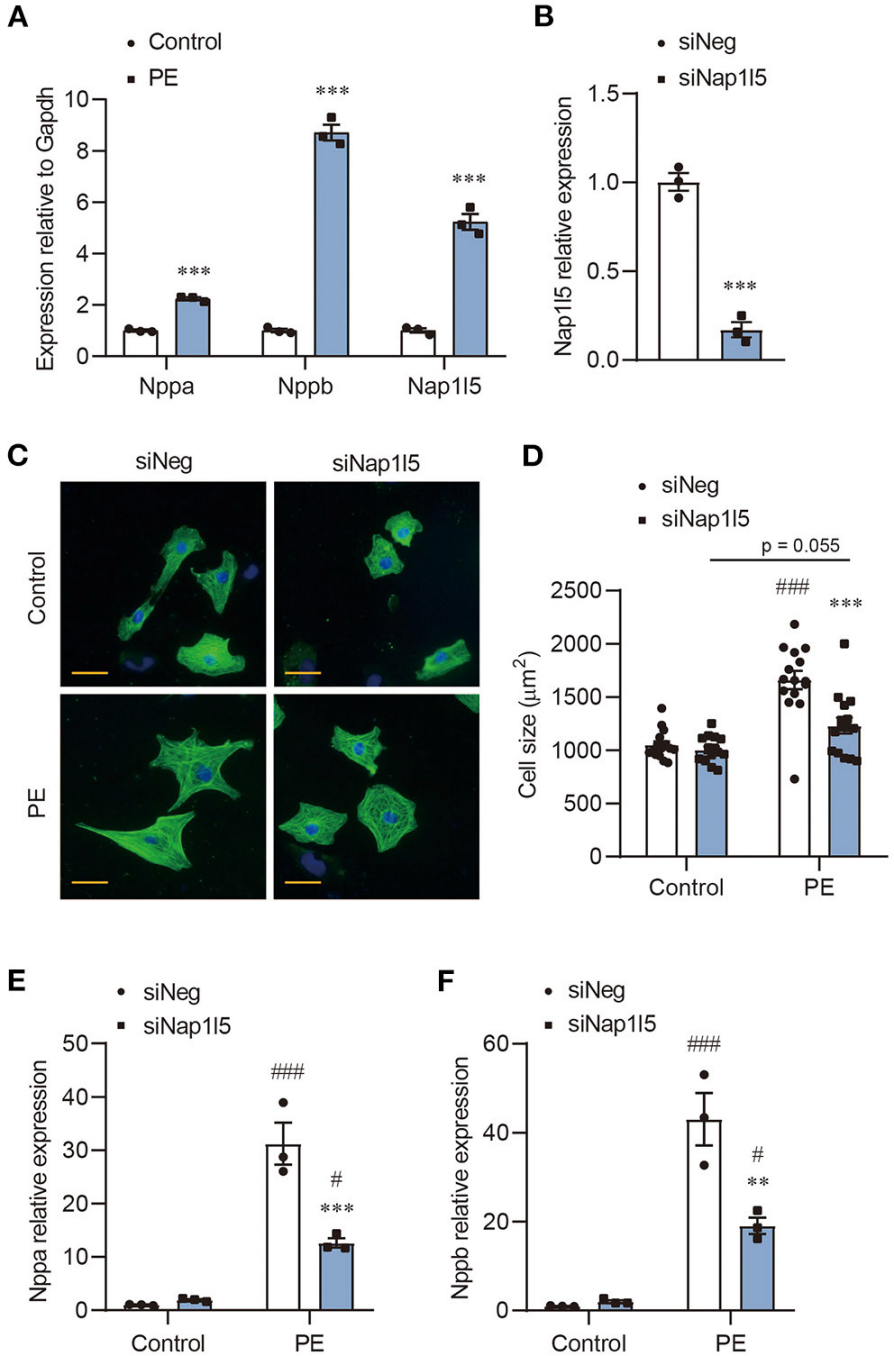
NAP1L5 silence abolishes PE-induced cardiomyocyte hypertrophy. **(A)** Expression of Nppa, Nppb and Nap1l5 in neonatal rat ventricular myocytes (NRVMs) with and without phenylephrine (PE; 50μM) treatment measured by qRT-PCR. ***P < 0.001 vs. Control. *n* = 3. **(B)** Knockdown efficiency of Nap1l5 expression by siNap1l5 in NRVMs measured by qRT-PCR. ***P < 0.001 vs. negative control siRNA (siNeg). *n* = 3. **(C)** Representative α-actinin-staining images of NRVMs transfected with siNeg or siNap1l5, with and without PE treatment. Scale bar: 20 μm. **(D)** Quantification of cell size measured by WGA staining in NRVMs transfected with siNeg or siNap1l5, with and without PE treatment. ***P < 0.001 vs. siNeg; ^###^*P* < 0.001 vs. Control. *n* = 15. **(E,F)** Impact of Nap1l5 knockdown on PE-induced expression of Nppa **(E)** and Nppb **(F)**. **P < 0.01, ***P < 0.001 vs. siNeg; ^#^*P* < 0.05, ^###^*P* < 0.001 vs. Control. *n* = 3.

We previously performed an RNA-seq analysis in PE-induced cardiomyocyte hypertrophy (17). After re-evaluating the genes with altered expression, we noticed a five-fold upregulation of Nap1l5, but not other Nap1l family members, in PE-treated NRVMs compared with control, which was validated by qRT-PCR (Figure 1A and Supplementary Figure 1A). To examine whether NAP1L5 plays a role in cardiomyocyte hypertrophy, we designed a Nap1l5-specific siRNA (siNap1l5) that achieving 80% knockdown efficiency 48h after transfection in NRVMs (Figure 1B and Supplementary Figure 1B). NAP1L5 silence did not change the cell size at basal level, but significantly suppressed PE-induced cell size enlargement (Figures 1C,D). Consistently, NAP1L5 knockdown significantly reversed the induction of hypertrophy markers, natriuretic peptide A (Nppa; also known as ANP) and natriuretic peptide B (Nppb; also known as BNP), after PE treatment (Figures 1E,F). These data suggest that NAP1L5 is required for cardiomyocyte pathological growth induced by PE.

### NAP1L5 Overexpression Promotes PE-Induced Cardiomyocyte Hypertrophy

**Figure 2:**
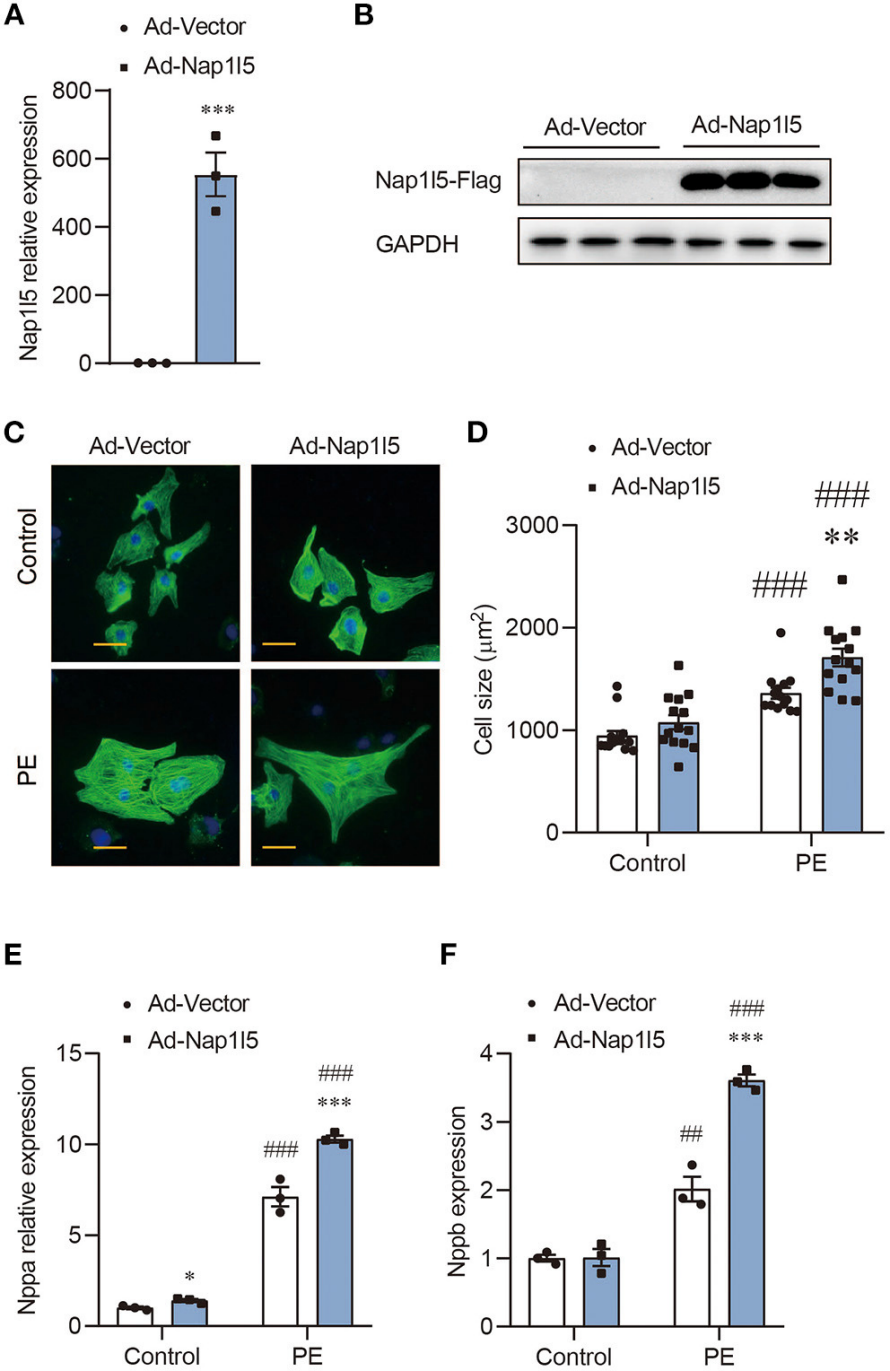
NAP1L5 overexpression promotes PE-induced cardiomyocyte hypertrophy. **(A)** Expression of Nap1l5 in NRVMs infected with adenovirus vector (Ad-Vector) or that expressing rat Nap1l5 (Ad-Nap1l5) measured by qRT-PCR. ***P < 0.001 vs. Ad-Vector. *n* = 3. **(B)** Immuoblots showing the validation of NAP1L5 overexpression at the protein level using an anti-FLAG antibody. GAPDH was used as an internal control. *n* = 3. **(C)** Representative α-actinin-staining images of NRVMs infected with Ad-Vector or Ad-Nap1l5, with and without PE treatment. Scale bar: 20 μm. **(D)** Quantification of cell size measured by WGA staining in NRVMs infected with Ad-Vector or Ad-Nap1l5, with and without PE treatment. **P < 0.01 vs. Ad-Vector; ^###^*P* < 0.001 vs. Control. *n* = 15. **(E,F)** Impact of Nap1l5 overexpression on PE-induced expression of Nppa **(E)** and Nppb **(F)**. **P < 0.01, ***P < 0.001 vs. siNeg; ^#^*P* < 0.05, ^###^*P* < 0.001 vs. Control. *n* = 3.

We then constructed an Adenovirus carrying rat Nap1l5 gene (Ad-Nap1l5) to overexpress it in NRVMs. Both qRT-PCR and Western blot analyses confirmed the overexpression of NAP1L5 48h after infection (Figures 2A,B). NAP1L5 overexpression had a marginal effect on cell size at basal level; however, it significantly aggravated the cell size enlargement after PE treatment (Figures 2C,D). Moreover, the PE-induced expressions of Nppa and Nppb were significantly enhanced by NAP1L5 overexpression (Figures 2E,F). These results indicate that NAP1L5 functions as a pro-hypertrophic factor.

### NAP1L5 Knockdown Reverses Hypertrophic Transcriptome Reprogramming

**Figure 3:**
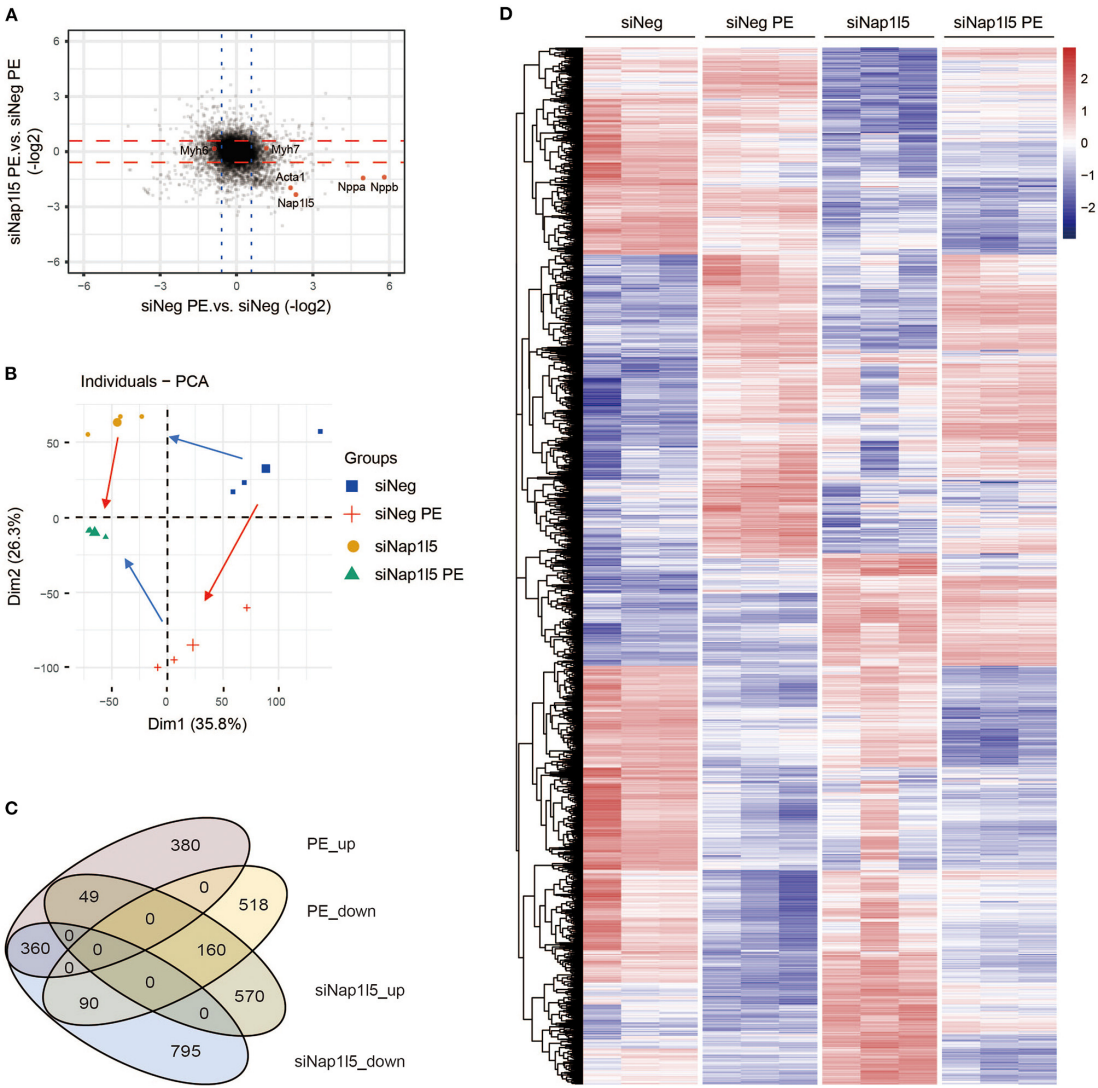
NAP1L5 knockdown reverses hypertrophic transcriptome reprogramming. **(A)** Two-dimensional plotting showing the impact of Nap1l5 knockdown and PE treatment on gene expression. Red and blue lines indicate the threshold of 1.5-fold changes. The expressions of Nap1l5, Nppa, Nppb, Acta1, Myh6, and Myh7 are highlighted. **(B)** Principal component analysis (PCA) showing the distribution of gene profiles from NRVMs transfected with siNeg or siNap1l5, with and without PE treatment. Red arrows indicate the changes from Control to PE; blue arrows indicate changes from siNeg to siNap1l5. **(C)** Venn diagram showing the relationship among up- and down-regulated genes by PE treatment or Nap1l5 knockdown. **(D)** Heatmap of the altered genes by either PE treatment or Nap1l5 knockdown.

To explore the underlying mechanism, we performed a transcriptome analysis in NRVMs with NAP1L5 knockdown. Expression plotting confirmed the knockdown of NAP1L5 and the suppression of hypertrophy markers, including Nppa, Nppb and Acta1 (actin alpha 1, skeletal muscle), after siNap1l5 transfection (Figure 3A). Principal component analysis (PCA) showed that NAP1L5 knockdown alleviated the pro-hypertrophic effect of PE treatment at the transcriptome level, and also caused a special impact on global gene expression (Figure 3B). Venn diagram showed that the down-regulated genes after NAP1L5 knockdown shared more genes with the upregulated genes after PE treatment in comparison with the down-regulated ones, and *vice versa* (Figure 3C), suggesting a general anti-hypertrophic impact of NAP1L5 silence on PE-induced transcriptome reprogramming. Furthermore, heatmap of the union set between siNap1l5-sensitive and PE-sensitive genes showed that NAP1L5 knockdown was generally oppositely correlated with PE in gene expression patterns (Figure 3D). These data further support a crucial role of NAP1L5 in pro-hypertrophic transcriptome reprogramming.

### NAP1L5 Does Not Affect Histone Methylation-Mediated Epigenetic Regulations

**Figure 4:**
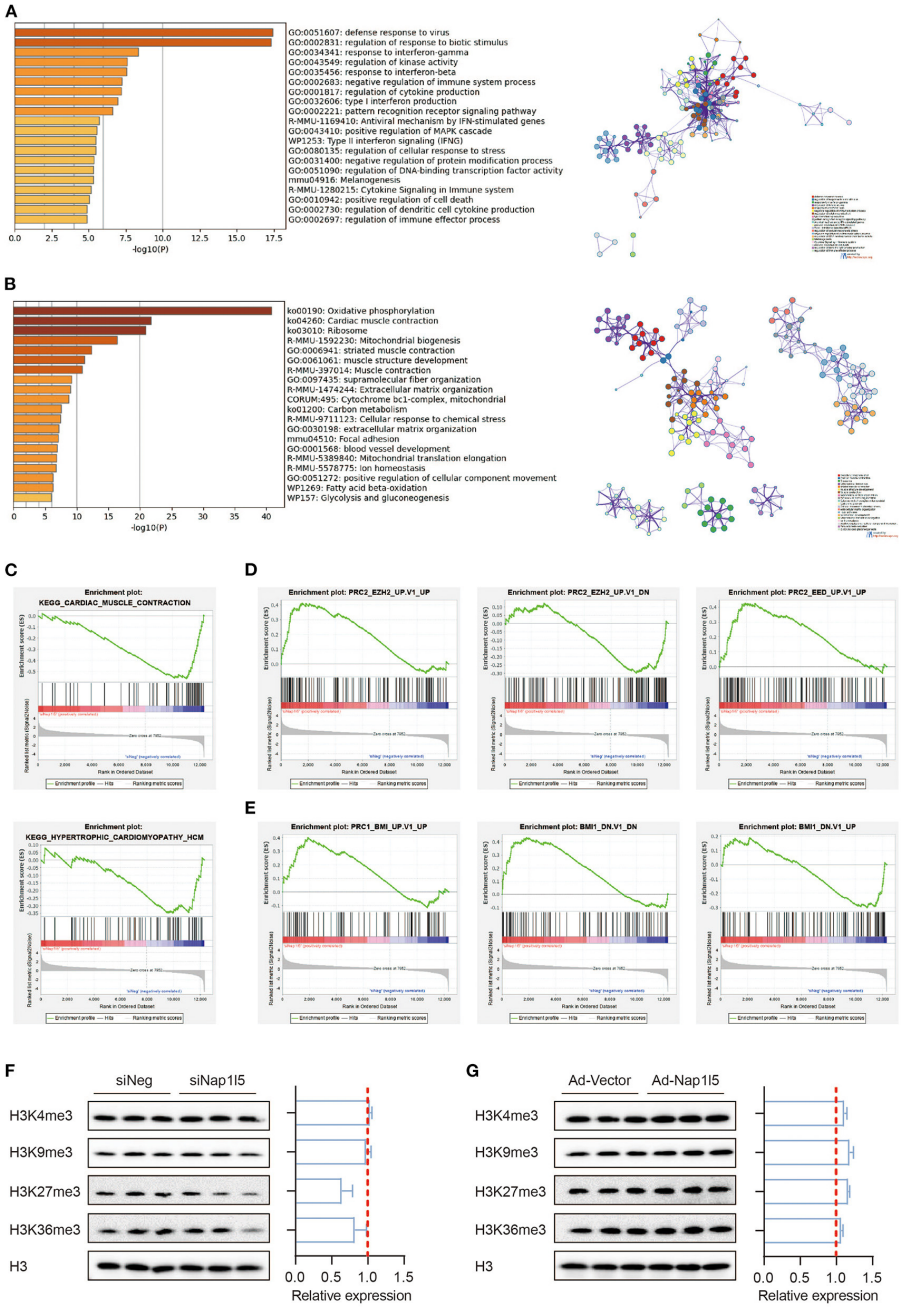
NAP1L5 does not affect histone methylation-mediated epigenetic regulations. **(A,B)** GO analysis (left) and protein-protein interaction (PPI; right) of the upregulated **(A)** and down-regulated **(B)** genes after Nap1l5 knockdown. **(C–E)** Gene set enrichment analysis (GSEA) showing the enrichment pattern of pathways, including cardiac muscle contraction (**C**; upper), hypertrophic cardiomyopathy (**C**; lower), PRC2_EZH2_UP (**D**; left), PRC2_EZH2_DOWN (**D**: middle), PRC2_EED_UP (**D**; right), PRC1_BMI_UP (**E**; left), PRC1_BMI_DOWN (**E**; middle), and BMI1_DOWN (**E**; right) after Nap1l5 knockdown. **(F,G)** Immunoblots (left) and quantification data (right) showing the impact of Nap1l5 knockdown **(F)** and Nap1l5 overexpression **(G)** on histone methylations at H3 K4, K9, K27, and K36 sites. *n* = 3.

Gene ontology (GO) analysis showed that the upregulated genes after NAP1L5 knockdown mainly related to immune responses, whereas the down-regulated genes covered oxidative phosphorylation, cardiac muscle contraction, and ribosome (Figures 4A,B). Gene set enrichment analysis (GSEA) also showed that hypertrophic cardiomyopathy-related genes were enriched in the down-regulated genes after NAP1L5 knockdown (Figure 4C), suggesting an involvement of NAP1L5 in hypertrophic cardiomyopathy.

Interestingly, the genes altered by siNap1l5 were negatively correlated with the regulations by polycomb repressive complex II (PRC2; Figure 4D) and complex I (PRC1; Figure 4E). We became curious about a possible role of NAP1L5 in epigenetic reprogramming. However, neither NAP1L5 knockdown nor its overexpression affected global methylations at histone H3 K4, K9, K27 and K36 sites, modifications essential for transcription regulation (Figures 4F,G). These data implicate that epigenetic regulation might not be the key mechanism underlying NAP1L5-mediated gene regulation.

### NAP1L5 Accelerates Protein Synthesis Rate

**Figure 5:**
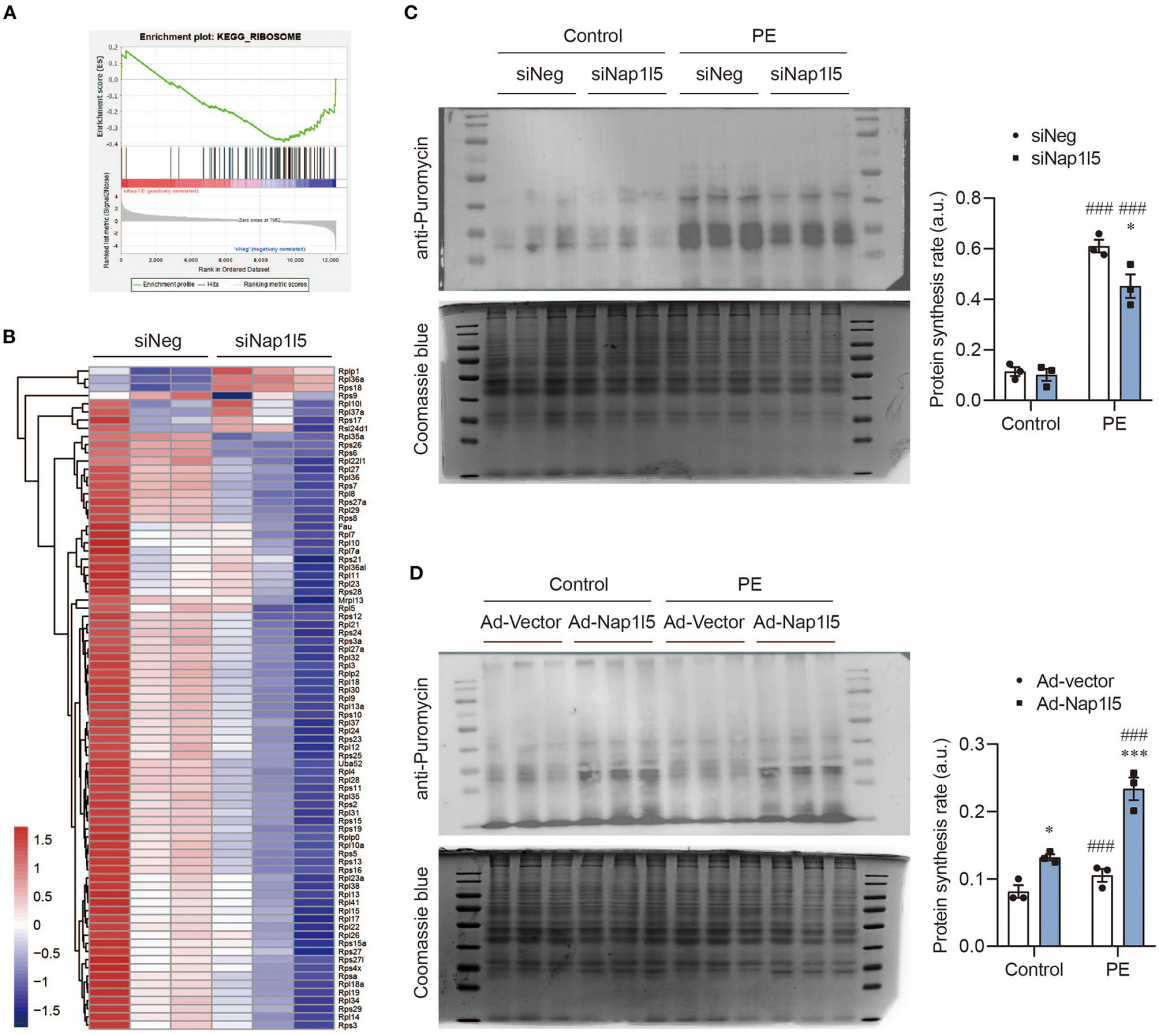
NAP1L5 accelerates protein synthesis rate. **(A)** GSEA showing the negative enrichment of ribosome-related genes after Nap1l5 knockdown. **(B)** Heatmap showing the expression of ribosome-related genes after Nap1l5 knockdown. **(C)** Puromycin incorporation assay showing the protein synthesis rate regulated Nap1l5 knockdown in NRVMs with and without PE treatment. Quantification data were normalized to Coomassie blue staining. *P < 0.05 vs. siNeg; ^###^*P* < 0.001 vs. Control. *n* = 3. **(D)** Puromycin incorporation assay showing the protein synthesis rate regulated Nap1l5 overexpression in NRVMs with and without PE treatment. *P < 0.05, ***P < 0.001 vs. siNeg; ^###^*P* < 0.001 vs. Control. *n* = 3.

Consistently with the GO analysis results, GSEA also revealed a down-regulation of ribosomal genes after NAP1L5 knockdown (Figure 5A), covering nearly all the genes of large and small ribosome subunits (Figure 5B). This result implicates a potential role of NAP1L5 in translation control. To test this hypothesis, we performed the puromycin incorporation assay, which allowing the detection of nascently synthesized peptides using Western blot with a specific anti-puromycin antibody (17, 44). We found that NAP1L5 knockdown significantly blocked the accelerated protein synthesis rate after PE treatment (Figure 5C). On contrary, NAP1L5 overexpression was sufficient to increase the protein synthesis rate (Figure 5D), suggesting that NAP1L5 promotes translation activity during cardiomyocyte hypertrophy.

### NAP1L5 Promotes Nucleolar Hypertrophy and Ribosome Assembly

**Figure 6:**
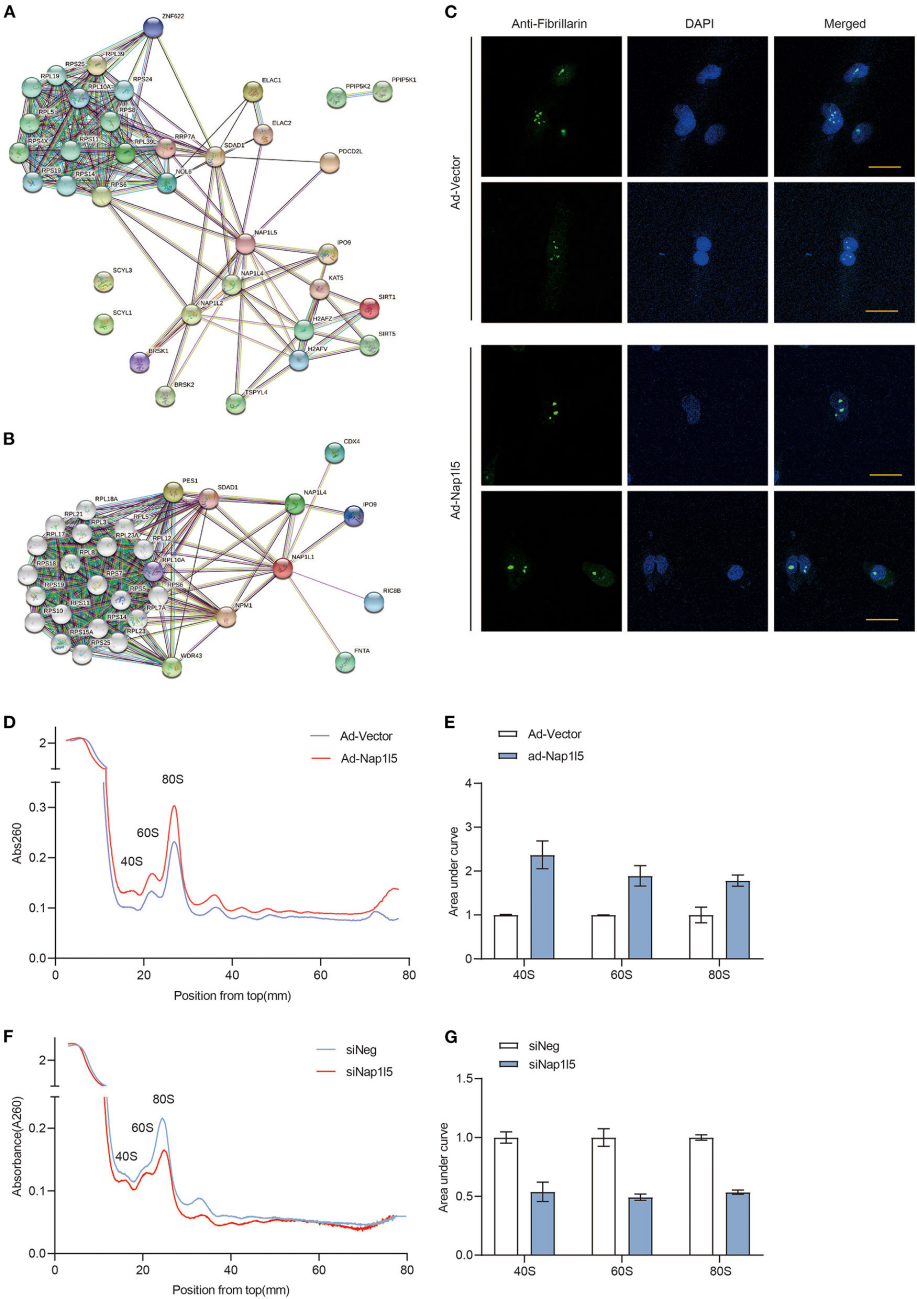
NAP1L5 promotes nucleolar hypertrophy and ribosome assembly. **(A)** Protein-protein interaction (PPI) network of NAP1L5 organized from String database. **(B)** PPI network of NAP1L1 organized from String database. **(C)** Representative nucleolar immunofluorescence images using anti-Fibrillarin antibody in NRVMs infected with Ad-Vector or Ad-Nap1l5. *n* = 15. **(D)** Ribosome profiles from NRVMs infected with Ad-Vector or Ad-Nap1l5 using sucrose gradient and detection of absorbance at 260nm. *n* = 2. **(E)** Quantification of 40S, 60S and 80S ribosomes from the ribosome profiling assay of panel **(D)**. Data were mean ± SD. *n* = 2. **(F)** Ribosome profiles from NRVMs transfected with siNeg or siNap1l5 using sucrose gradient and detection of absorbance at 260 nm. *n* = 2. **(G)** Quantification of 40S, 60S and 80S ribosomes from the ribosome profiling assay of panel **(F)**. Data were mean ± SD. *n* = 2.

We next analyzed the interactome of NAP1L5 from the String database, and found that NAP1L5 linked to core ribosomal proteins through binding to ribosome assembly or transport factors, including SDAD1, RRP7A, and NOL6 (Figure 6A). Interestingly, NAP1L1 also exhibited a similar interactome covering most of the NAP1L5-interacting factors (Figure 6B), although they share little identity in protein sequence (22.5%) or structure (Supplementary Figure 2). These observations implicate that NAP1L5 might participate in ribosome assembly. Nucleolus develops hypertrophy to accelerate ribosomal RNA transcription and ribosome assembly upon pro-growth stimulation (45). We found that NAP1L5 overexpression substantially increased the size of nucleolus, as evidenced by immunofluorescence of a nucleolar marker Fibrillarin (Figure 6C) (46). We then examined the ribosome profiles in NRVMs with NAP1L5 overexpression or knockdown. Compared with the Ad-Vector control, Ad-NAP1L5-infected NRVMs exhibited more ribosomal contents in the 40S, 60S, and 80S components (Figures 6D,E). Consistently, Nap1l5 knockdown in NRVMs reduced the contents of 40S, 60S and 80S components (Figures 6F,G), suggesting a crucial role of NAP1L5 in ribosome biogenesis.

To further explore the molecular mechanism, we tried to validate the interaction between NAP1L5 and SDAD1 or NOL6. Unfortunately, we did not observe a direct interaction between NAP1L5 and SDAD1, nor the full-length or spliced NOL6 isoforms (Supplementary Figure 3). These data suggest that other factors may be involved in the regulation of ribosome assembly by NAP1L5.

## Discussion

The pathogenesis of cardiac hypertrophy is a programmed process mediated by regulations at multiple layers such as transcription, translation, and metabolism (2, 5, 47–49). Compared with other aspects, our knowledge about translation control during cardiac hypertrophy is relatively limited. Here we find that NAP1L5 plays a key role in PE-induced cardiomyocyte hypertrophy through promoting nucleolar hypertrophy, ribosome assembly and protein synthesis.

Ribosome biogenesis is a highly energy-consuming process. After translation, ribosomal proteins need to be transported into nucleus and precisely folded with mature rRNA inside nucleolus (50). Cardiac hypertrophy is one of the first diseases identified to be associated with ribosomal DNA transcriptional disorders (8, 9). Hypertrophied cardiomyocyte is characterized by excess protein synthesis to meet the increased demand for cell function maintenance, which necessitates an accelerated ribosome biogenesis (8, 11, 51–53). Accelerated PolI transcription activity increases ribosome numbers during the development of cardiac hypertrophy (10–12). Beyond these observations, we know little about the other processes related to ribosome life cycle. Our discovery about the role of NAP1L5 in translation control accounts for a novel step toward the dynamic regulation of ribosome assembly under pathological conditions.

During evolution, higher ordered plants and mammals have acquired several paralogues of NAP1, named NAP1 like family, and five different NAP1L proteins (NAP1L1-5) have been identified. The overall structure of NAP1L proteins is highly conserved and the protein sequences of human NAP1L homologs to hNAP1L1 show identities ranging between 31% for hNAP1L5 and 64% for hNAP1L4 (54). These NAP1L proteins all contain a NAP1L motif, which is positioned within their central domain and critical for their histone chaperone activity (55). As the newest identified member of the NAP1L family, NAP1L5 has the shortest amino acid sequence (182 aa) among NAP1L family members, and its function has not been elucidated yet. Nevertheless, its homolog NAP1L1 and NAP1L2 have been reported to be histone chaperones, assisting the assembly and disassembly of nucleosome at active transcription sites (21, 23, 56–58). Okuwaki et al. (57) found that NAP1L1 was specifically responsible for the assembly and disassembly of H2A-barr body deficient variant. Tachiwana et al. (58) found that NAP1L2, but not NAP1L1, was required for the incorporation of testis-specific H3t variant into nucleosome. Attia et al. (59) reported that all five members of NAP1L family were able to interact with each other directly via their highly conserved alpha helices. Our analyses from the String database also revealed that NAP1L5 might directly interact with NAP1L2 and NAPL4 (Figure 6A). A series of translatome-scale affinity capture studies identified the direct interaction between NAP1L1 and NAP1L2 (60–63). Thus, our findings indicate that the NAP1L proteins might form a special complex involving in the nucleosome dynamics during rRNA transcription.

SDAD1 (SDA1 domain containing 1) is a shared hub protein interacting with both NAP1L1 and NAP1L5 (Figures 6A,B). It has been involved in the development of cardiac hypertrophy and tumor diseases through binding with long non-coding RNAs or microRNAs (64–66). Interestingly, the yeast homolog of SDAD1 has been shown to interact with NAP1 and facilitates the export of 60S pre-ribosomal subunits from nucleus to cytoplasm (67, 68). NOL6 is a highly conserved nucleolar protein that appears to be associated with ribosome biogenesis by interacting with pre-rRNA primary transcript (69). Although interactions of NAP1L5 with SDAD1 and NOL6 have been implicated by affinity capture mass spectrum, we could not validate their direct interactions using co-IP assay (Supplementary Figure 3). Whether and how NAP1L5 links to the ribosome assembly process and contributes to the pathogenesis of cardiac hypertrophy need to be further investigated.

Regulation of cell growth is a fundamental process in development and disease that integrates a vast array of extra- and intra-cellular information. A central player in this process is PolI, which transcribes ribosomal RNA (rRNA) genes in the nucleolus. Rapidly growing cancer cells are characterized by increased Pol I-mediated transcription, and consequently nucleolar hypertrophy. An aberrant increase in nucleolar size in cancer cells was documented more than a century ago. Nucleolar hypertrophy is a common feature in cancer and that nucleolar size can be used as a histopathological marker to grade the malignancy of tumors (70, 71). Despite this early association of enlarged nucleoli and cancer, little is known about nucleolar size in cardiac hypertrophy. Pathological growth of cardiomyocytes during hypertrophy is characterized by excess protein synthesis. Our data provide crucial evidence that overexpression of Nap1l5 aggravates cardiac hypertrophy along with increased ribosome assembly and translation activity. This might be attributable to its impact on PolI-mediated transcription and subsequently nucleolar hyperplasia.

mTOR is at the core of the translational regulation by converging signaling transduction from a variety of nutrients and growth factors (13, 14, 72–76). Activation of mTOR signaling has been involved in the pathogenesis of cardiac hypertrophy; however, cardiac-specific ablation of raptor impairs adaptive hypertrophy, but causes heart failure in mice (15). Moreover, simultaneous knockout of two genes encoding for S6K1, Rps6kb1 and Rps6kb2, has no effect on pressure overload-induced cardiac hypertrophy in mice (16). Interestingly, our previous study implicated that the activation of mTOR signaling is usually transient, and quickly fades out after hypertrophic stimulation (17). Due to the crucial role of mTOR in cell survival and the diverse outcomes of its inactivation (15, 16), mTOR would not be a good drug target for cardiac hypertrophy therapy. Our findings suggest that the mTOR-independent translation control might contribute even more to the accelerated protein synthesis during cardiac hypertrophy.

One limitation of this study is that all observations are from an *in vitro* cellular model induced by an α1-receptor agonist, PE. Whether this mechanism also applies *in vivo* and in human systems need to be further investigated.

## Conclusions

Taken together, we demonstrate that NAP1L5 is upregulated in PE-induced cardiomyocyte hypertrophy, which functions to promote protein synthesis through facilitating ribosome assembly. Our findings provide novel insights into the translation control during cardiac hypertrophy, and provide potential molecular targets to treat cardiac hypertrophy and heart failure.

## Methods

### Cell Culture

Neonatal rat ventricular myocytes (NRVMs) were isolated and cultured as described previously (17). Neonatal rat hearts (within 3 days after birth) were immediately extracted after decapitation and placed in a dish containing precooled PBS. Left ventricular tissues were dissected, finely cut into pieces, and then digested in a solution containing 0.08% type II collagenase (Sigma) and 0.125% protease (Sigma) at 37°C for 20 min/time. The supernatants were discarded at the first time and collected after that. This process continued until the heart tissues were completely digested. Cardiomyocytes were separated from fibroblasts by percoll (GE) density gradient centrifugation, and cultured in high glucose DMEM (Hyclone) containing 10% FBS (Gbico) and 1% penicillin/streptomycin. After 24 h of culture, the medium was changed to DMEM medium with 1% ITS (Invitrogen) for another 24 h before further treatment.

### Plasmids

The full-length cDNA sequence of rat Nap1l5 (rNap1l5) were obtained by the National Center for Biotechnology Information (NCBI). The pcDNA3.1-rNap1l5-Flag recombinant plasmid were constructed by cloning the entire coding region of rNap1l5 into the NheI and XhoI sites of the pcDNA3.1-HA plasmid. The primer pair rNap1l5 CZF (5′- GGGAGACCCAAGCTGGCTAGC*GCCACC*atggccgaccccgagaag−3′) and rNap1l5 CZR (5′- TACGTCGTATGGGTA*TCTAGA*Tcttctcggcagagtcgggacc−3′) was used to amplify a cDNA fragment encoding the mature rNap1l5 peptide. Massive plasmid replication was performed by transforming the pcDNA3.1-rNap1l5-Flag plasmid into E. coli DH-5α. In addition, in order to overexpress Nap1l5 in NRVM, the pcDNA3.1-rNap1l5-Flag plasmid was sent to Shanghai Hanheng Biotechnology Co., Ltd. for adenovirus packaging.

The full-length cDNA sequence of rat Nol6 and SDAD1 were obtained by the National Center for Biotechnology Information (NCBI). The pcDNA3.1-SDAD1-HA recombinant plasmid were constructed by cloning the entire coding region of SDAD1 into the NheI and XhoI sites of the pcDNA3.1-Flag plasmid. The primer pair SDAD1 CZF (5′- *ACTATAGGGAGACCCAAGCTGGCTAGCGCCACCatgtccgggaggaacaac*−3′) and SDAD1 CZR (5′- *ATCTGGTACGTCGTATGGGTATCTAGActtcattcttttcctctttttcag*−3′) was used to amplify a cDNA fragment encoding the mature SDAD1 peptide. Again, to get the mature Nol6 peptide, the primer pair Nol6 CZF (5′- *ACTCACTATAGGGAGACCCAAGCTGGCTAGCGCCACCatgggaccagcccccgccgga*−3′) and Nol6 CZR (5′- *ATCTGGTACGTCGTATGGGTATCTAGAcacagtccacctctcacttcggg*−3′) was used to amplify a cDNA fragment encoding the mature Nol6 peptide. However, the sequencing results showed that the deletion of exon 18 of this Nol6 resulted in frameshift mutation after exon 18, so we named this spliced NOL6 isoforms as Nol6Δ. To get the full-length Nol6, we used genomic DNA of Nol6 as a template and designed primers NOL6 gBF (tggtttgtcacctggagggcagcgg) and NOL6 gBR (cttgaggacgtcagtgtgtgtggcag) amplified in the upstream and downstream of exon 18 to obtain the missing fragment, and then the-full length Nol6 was obtained by bridging and recombination.

### Cell Size Measurement

Cell size was measured using wheat germ agglutinin (WGA) staining as previously described. Briefly, cardiomyocytes were stimulated with 50 μM of PE for 48 h after transfection with different siRNA or infection with different adenoviruses for 24 h, the culture medium was discarded, washed with PBS, and fixed with 4% paraformaldehyde for 15 min at room temperature. Then 5.0 μg/mL WGA (Invitrigen) was applied to incubate with cardiomyocytes for 10 min at 37°C. When labeling was complete, removed the labeling solution, and washed cells three times with PBS. Then dyed with DAPI for 10 min at room temperature, and washed 5–7 times with PBS for 5 min each time. Finally, Images collected under a fluorescence microscope. Image Pro Plus 6.0 software was used to measure cardiomyocyte surface area. Each field of view measured more than 50 to calculate the surface area of cardiomyocytes.

### Immunofluorescence

The method of immunofluorescence staining of cardiomyocytes was as described above. The cardiomyocytes used for immunofluorescence staining were cultured in a six well cell culture plate containing sterile glass slides. Cardiomyocytes were stimulated with 50 μM of PE for 48 h after transfection with different siRNA or infection with different adenoviruses for 24 h, The cells were then fixed with 4% formaldehyde in PBS for 15 min at room temperature, permeabilized with 0.1% Triton X-100 in PBS for 10 min, goat serum was blocked at room temperature for 30 min, and then incubated with α-actinin antibody (1:100, Proteintech) or Fibrillarin antibody (1:100, Abclonal) at 4°C overnight. The next day, incubated the cell sample with the corresponding secondary antibody for 1 h at room temperature. Then dyed with DAPI for 10 min at room temperature. Then washed 5–7 times with PBS for 5 min each time. Finally, removed the slides from the 6-well plate and mounted the slides. Then, the fluorescence staining pictures of cardiomyocytes were collected under a fluorescence microscope. Image Pro Plus 6.0 software was used observe and take pictures.

### Immunoprecipitation

Cultured HEK293T cells co-transfected with the appropriate plasmids were collected and lysed in an IP buffer (50mM Tris-HCl (pH 8.0), 150mM NaCl, 2mM EDTA, 1% NP-40 and 5% Glycerol) supplemented with a protease inhibitor cocktail (Roche) and PMSF. After incubating for 30 min at 4°C, followed by centrifuging 13,000 g for 15 min, the cell lysates were precleared with normal mouse or rabbit immunoglobulin G and protein A/G-agarose beads (Roche) for 1h at 4°C. The precleared lysates (500 μl) were then incubated with 1 μg of antibody and 10 μl of protein A/G-agarose beads on a rocking platform at 4°C overnight. The immunocomplex was collected, washed 5–6 times with cold IP wash buffer (50mM Tris-HCl (pH 8.0), 150mM NaCl, 2mM EDTA, 0.1% NP-40) and blotted using the indicated primary antibodies.

### Western Blot

Total proteins were extracted from NRVM cells. NRVM cells lysed in Radio Immunoprecipitation Assay (RIPA) lysis buffers (Beyotime, Nanjing, China). Cell lysates were centrifuged at 12,000 × g for 15 min. The proteins concentration was detected by the BCA method, and equal quantities of protein extracts were loaded on a sodium dodecyl sulfate-polyacrylamide gel electrophoresis (SDS-PAGE), then transferred onto polyvinylidene fluoride (PVDF) membranes (Millipore Corp., Bedford, MA, U.S.A.). The membranes were blocked with 5% (w/v) non-fat milk for 1 h at room temperature, the membranes were incubated with specific primary antibodies overnight at 4°C. After washing 3 times with TBST, the membranes were incubated with the HRP-linked secondary antibody at room temperature for 1 h, and then washed 3 times with TBST again. Finally, the protein bands on the membranes were detected by chemiluminescent reagents (Beyotime, Nanjing, China). Chemiluminescence signals were quantified using an ECL imager, and analyzed using Quantity One software (Bio-Rad, Hercules, CA, USA). The specific primary antibodies were: anti-GAPDH (Proteintech), anti-Histone3 (Proteintech), anti-H3K4me3 (CST), anti-H3K9me3 (CST), anti-H3K27me3 (CST), anti-H3K36me3 (CST), anti-Flag (Sigma), and anti-Puromycin (Santa Cruz).

### Puromycin Incorporation Assay

Different treatments of NRVM cells, 30 min before harvesting, add 1μM puromycin to each well for 30 min, then harvest the cells and extract the protein, and detect the binding level of puromycin by Western blot with anti-Puromycin antibody.

### Ribosome Profiling

Ribosome profiling was performed as described. Cardiomyocytes were transfection with different siRNA or infection with different adenoviruses for 48 h, and were treated with with CHX (100 μg/mL) for 10–30 min before harvested. Cells were harvested using trypsin and then lysed in polysome extraction buffer (20 mM Tris-HCl [pH 7.0], 100 mM KCl, 5 mM MgCl_2_, 0.5% Nonidet P-40) containing 100 μg/ml CHX, 1x protease inhibitors and 1:1,000 dilution of RiboLock RNase inhibitor for 15 min on ice. Following centrifugation, lysates were ultracentrifuged on 10–50% sucrose gradients at 190,000g for 1.5 h at 4°C. Following ultracentrifugation, fractions were collected from each sample using a BioComp Piston Gradient Fractionator instrument fitted with a TRIAX flow cell to measure absorbance.

### RNA-Seq Analysis

NRVMs were transfected with siNap1l5 or siNeg using Lipofectamine RNAiMAX. After transfected 24 h, 50 μM phenylephrine (PE) were added in the media to induce cardiomyocyte hypertrophy, was performed as described previously (17). 48 h after treated with PE, NRVMs were harvested to extracted RNA according to the manufacturer's instructions. Transcriptome sequencing of RNA was completed by Beijing Genomics Institution (BGI). Three independent biological replicate samples were sequenced for each group. (i) The different expression genes (DEGs) between groups were screened using linear models for microarray data (limma) package in R. |Fold change| >1.5 and adjusted *P*-value < 0.05 were considered the threshold. (ii) Enrichment analysis of DEGs using Metascape online database with default parameters. (iii) Gene Set Enrichment Analysis (GSEA) is used to screen significantly enriched signaling pathways and transcription factors with default parameters, was performed as described previously.

### Quantitative Real-Time PCR

Total RNA was extracted from NRVM cells using the GenElute Mammalian Total RNA Miniprep Kit (Sigma-Aldrich), following the manufacturer's instructions. RNA was quantified using NanoDrop (Thermo Fisher Scientific). The cDNAs were synthesized from 1μg RNA using a RevertAid First Strand cDNA Synthesis Kit (Thermo Fisher Scientific, U.S.A.). Real-time PCR was performed using the specific primers and Ultra SYBR Mixture (Monad, Suzhou, China) on CFX96M Touch Real-Time PCR Detection System (Roche, Basel, Switzerland). The PCR primer sequences used in this study include rNap1l5-forward: GAGCACAGCAGCTGACAGAC; rNap1l5-reverse: ATGACGTCGTTCTTGGGTTC; rNppa-forward: ATACAGTGCGGTGTCCAACA; rNppa-reverse: AGCCCTCAGTTTGCTTTTCA; rNppb-forward: CAGCTCTCAAAGGACCAAGG; rNppb-reverse: GCAGCTTGAACTATGTGCCA; rGAPDH-forward: ACAGCAACAGGGTGGTGGAC; rGAPDH-reverse: TTTGAGGGTGCAGCGAACTT. Relative expression of genes was determined with GAPDH as an endogenous control.

### Quantification and Statistical Analysis

Statistical analyses were performed using GraphPad Prism 8 Software. All experimental data are presented as mean ± SEM of at least three independent experiments unless denoted elsewhere. Statistical significance for multiple comparisons was determined by one-way ANOVA or two-way ANOVA followed by Tukey's test. Bonferroni adjustment was used for *post hoc* analysis. Student's *t*-test was used for comparisons between two groups. *P* < 0.05 was considered statistically significant.

## Data Availability Statement

All relevant data supporting the findings of this study are available within the article and its supplementary information or from the authors upon reasonable request. RNA-seq data have been deposited in NCBI's Gene Expression Omnibus (GEO) repository (accession code GSE173737).

## Ethics Statement

The animal study was reviewed and approved by Renmin Hospital of Wuhan University.

## Author Contributions

ZW and JT conceptualized, designed, and supervised the study. NG, DZ, and JS performed the molecular and cellular experiments with inputs from SW. JL performed the bioinformatic analyses. YF, SZ, and QG assisted with the preparation of NRVMs. ZW and NG analyzed the data and wrote the manuscript. All authors have approved its publication.

## Funding

This study was supported by funds from National Natural Science Foundation of China (Nos. 81722007 and 82070231), National Health Commission of China (No. 2017ZX1030440 2001-008), State Key Laboratory of Cardiovascular Disease (Nos. GZ2021015 and 2021-YJR01), and start-up funds from Fuwai Hospital Chinese Academy of Medical Sciences, Shenzhen (GSP-RC-2020002).

## Conflict of Interest

The authors declare that the research was conducted in the absence of any commercial or financial relationships that could be construed as a potential conflict of interest.

## Publisher's Note

All claims expressed in this article are solely those of the authors and do not necessarily represent those of their affiliated organizations, or those of the publisher, the editors and the reviewers. Any product that may be evaluated in this article, or claim that may be made by its manufacturer, is not guaranteed or endorsed by the publisher.

## Abbreviations

4E-BP1: eIF4E-binding protein-1
Acta1: Actin alpha 1
CHX: Cycloheximide
DAPI, 4': 6-Diamidino-2-phenylindole
DEGs: The different expression genes
eIF4E: Eukaryotic translation initiation factor 4E
GEO: Gene Expression Omnibus
GO: Gene ontology
GSEA: Gene set enrichment analysis
mTOR: The mechanistic target of rapamycin
NAP1L: Nucleosome assembly protein 1 like protein family
Nap1l5: Nucleosome assembly protein 1 like 5
NOL6: Nucleolar Protein 6
Nppa/ANF: Natriuretic peptide A
Nppb/BNP: Natriuretic peptide B
NRVMs: Neonatal rat ventricular myocytes
PCA: Principal component analysis
PE: Phenylephrine
PMSF: Phenylmethylsulfonyl fluoride
PolI: polymerase I
PRC1: Polycomb Repressive Complex 1
PRC2: Polycomb Repressive Complex 2
Rps6kb1: Ribosomal Protein S6 Kinase B1
Rps6kb2: Ribosomal Protein S6 Kinase B2
RRP7A: Ribosomal RNA Processing 7 Homolog A
S6K1: Ribosomal protein S6 kinase 1
SDAD1: SDA1 domain containing 1
UBTF: Upstream binding transcription factor
WGA: Wheat germ agglutinin.
