# Supplementary material for: NAP1L5 Promotes Nucleolar Hypertrophy and Is Required for Translation Activation During Cardiomyocyte Hypertrophy
Source: Front Cardiovasc Med. 2021 Dec 17;8:791501. doi: 10.3389/fcvm.2021.791501 (PMC8718910; doi:10.3389/fcvm.2021.791501)
Supplement: Supplementary file 1 [file Data_Sheet_1.PDF]

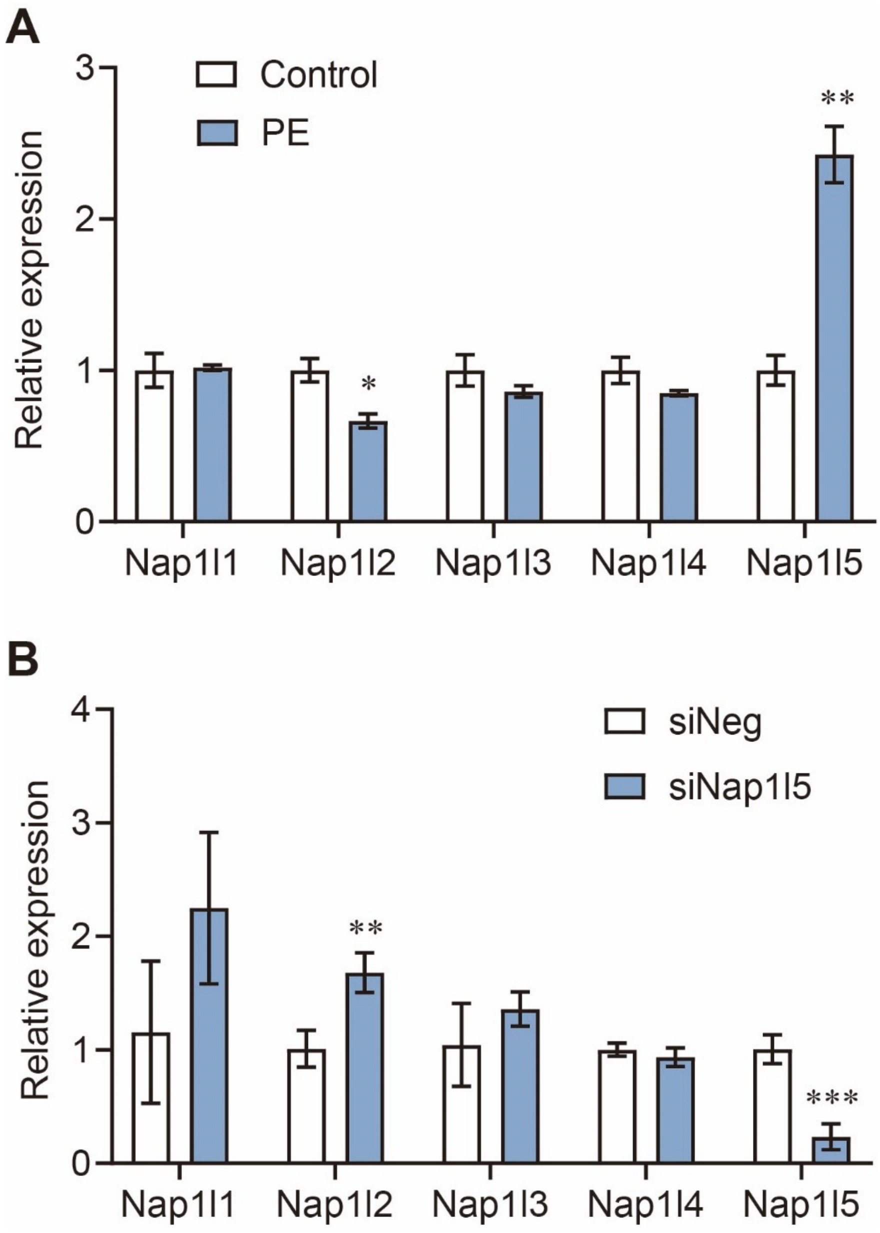

Supplementary Fig. 1. Validation of the expression levels of NAP1L family members in NRVMs.

(**A**) qRT-PCR analysis showing the impact of PE treatment on the mRNA levels of NAP1L family members. *P < 0.05, **P < 0.01 vs. Control. n = 3.

(**B**) Validation of the knockdown specificity of siNap1l5 in NRVMs. Protein domain analysis of NAP1L1-5. **P < 0.01, ***P < 0.001 vs. siNeg. n = 3.

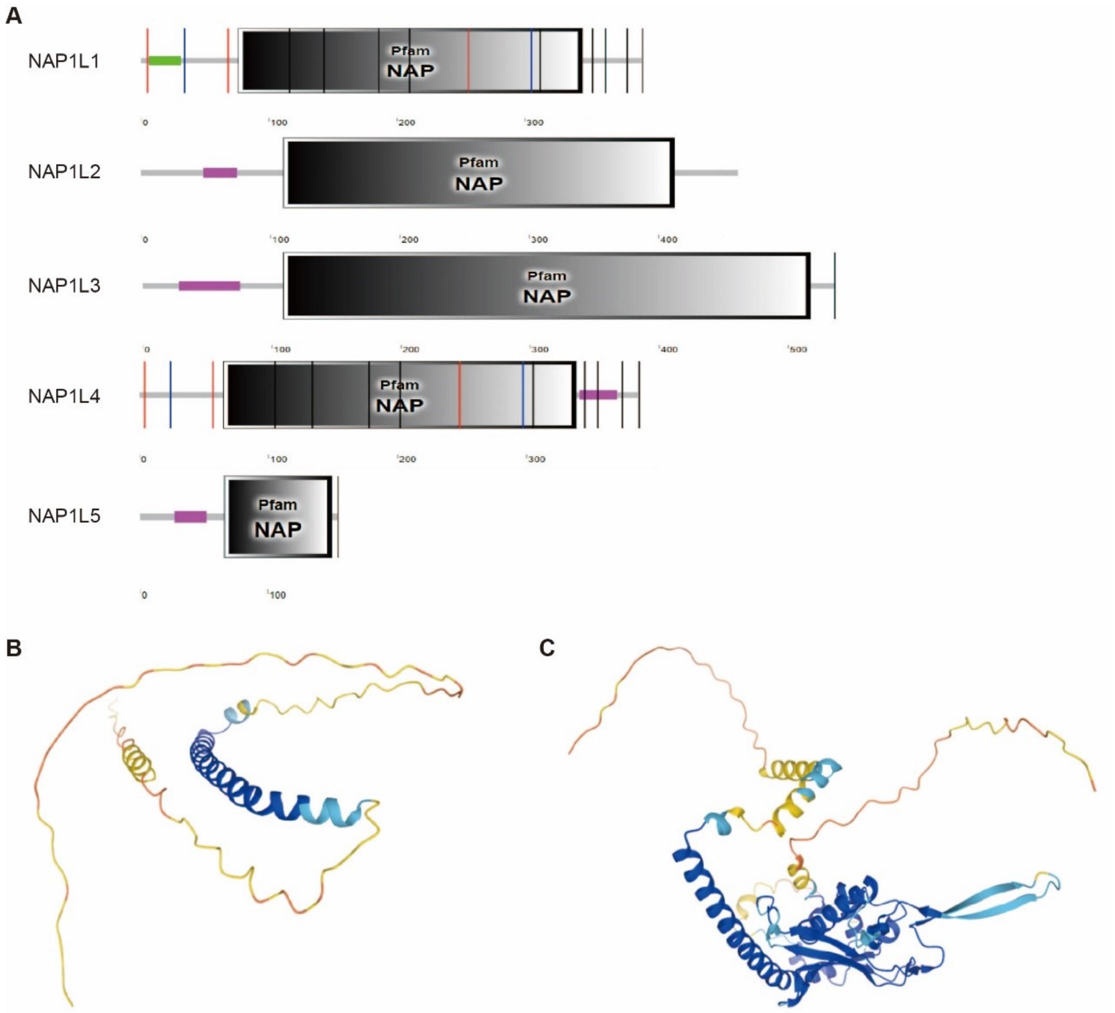

Supplementary Fig. 2. Sequence and structural conservation of NAP1L family members.

(**A**) Protein domain analysis of NAP1L1-5.

(**B**) NAP1L5 protein structure predicted by AlphaFold 2.0.

(**C**) NAP1L1 protein structure predicted by AlphaFold 2.0.

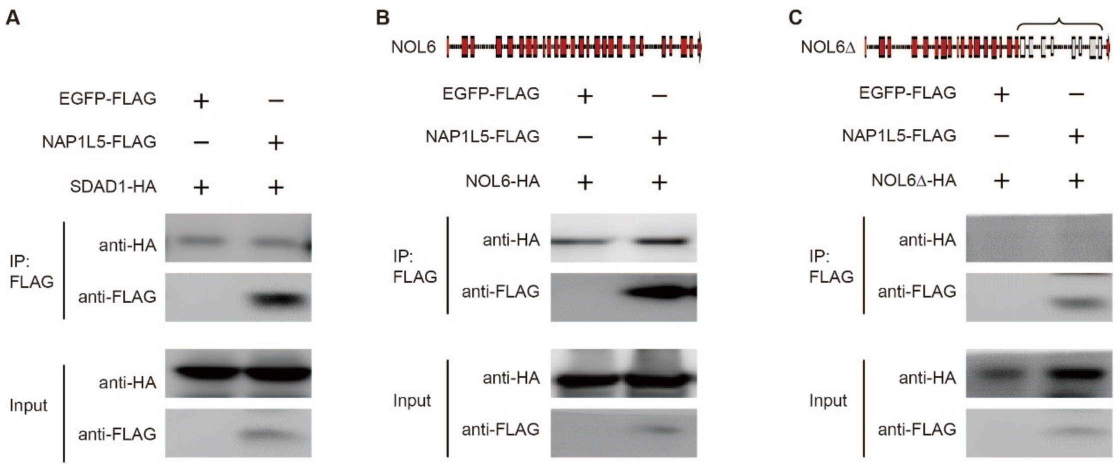

Supplementary Fig. 3. NAP1L5 does not directly interact with SDAD1 or NOL6.
(**A**) Co-IP assay showing the potential interaction between NAP1L5 (FLAG-tagged) and SDAD1 (HA-tagged).
(**B**) Co-IP assay showing the potential interaction between NAP1L5 (FLAG-tagged) and full-length NOL6 (HA-tagged).
(**C**) Co-IP assay showing the potential interaction between NAP1L5 (FLAG-tagged) and spliced NOL6 isoform (HA-tagged).
